# Haplotypes inside the beta-globin gene: use as new biomarkers for beta-thalassemia prenatal diagnosis in north of Iran

**DOI:** 10.1186/s12929-017-0396-y

**Published:** 2017-12-04

**Authors:** Mohammad Bagher Hashemi-Soteh, Seyed Saeed Mousavi, Alireza Tafazoli

**Affiliations:** 1 Immunogenetic Research Center, Molecular and Cell biology Research Center, Medical Faculty, Mazandaran University of Medical Sciences, Sari, Mazandaran Iran; 2 Novin Genetics diagnostic laboratory, Farah Abad 4, Farah Abad Boulevard, Sari, Mazandaran Iran; 3 Department of Biochemistry, Biophysics and Genetics, School of Medicine, Mazandaran University of Medical Sciences, Farahabad Road, P.O. Box. 481751665, Sari, Mazandaran Iran; 4 Medical Genetics Research Center, Mashhad University of Medical Sciences, Mashhad, Iran

**Keywords:** Beta-thalassemia, Beta-globin gene, Haplotypes, Prenatal diagnosis, Mazandaran

## Abstract

**Background:**

Beta-thalassemia is common in the Mediterranean area as well as the Middle East and India. Official report in Iran revealed the average prevalence rate of carriers about 4%. More than 20 restriction fragment length polymorphisms (RFLPs) are known in the beta-globin gene cluster and used in the prenatal diagnosis (PND) services. Some of these locations may have low allele frequency and are not informative in the prenatal diagnosis. The current study aims to find new haplotypes and polymorphisms with high allele frequency in the local population.

**Methods:**

Two thousand three hundred fifty samples (1,321 male and 1,029 female) from the northern Iran, whom suspected to be the carriers either for alpha or beta thalassemia and referred to the local diagnostic laboratory as a routine services were investigated during five years, (2010–2015). The beta-globin gene was sequenced for all samples.

**Results:**

Heterozygosity for five SNPs in the beta-globin gene was calculated separately. 383 individuals (16.29%) showed no sign of nucleotide change in the beta-globin gene sequence. In total, codon2 (C/T) 31.72%, IVSII-16 (C/G) 31.72%, IVSII-74 (G/T) 54.71%, IVSII-81 (C/T) 19.47%, and IVSII-666 (T/C) 31.72% were seen respectively. Although all five polymorphisms showed reasonably high heterozygosity, IVSII-74 (G/T) [GG wild type (36.5%), G/T (54.71%) and TT (8.8%)] revealed the highest heterozygosity rate. Four combinations of these five SNPs were defined as new haplotypes named M1 to M4. ARMS-PCR also were designed and applied to detect IVSII-74 (G/T) nucleotide position.

**Conclusions:**

This study represents an intragenic polymorphism, IVSII-74, a reliable position with high heterozygosity rates in Iranian population for PND analysis.

**Trial registration:**

Retrospectively registered.

## Background

Thalassemia is a highly prevalent genetic disorder across Asia and some parts of Europe. Beta-thalassemia is more common in the Mediterranean area as well as the Middle East and India [1, 2]. An official report from the Genetics Office in Iran’s Ministry of Health and Medical Education (GO-MOH) reveal that the average prevalence rate of thalassemia carriers in Iran is about 4%, varying at different provinces with the highest rate of 9.5% in Kerman and the lowest 1% in West Azerbaijan. Mazandaran, with a carrier rate of more than 8.0%, is also among the provinces with a high rate [3, 4]. According to the statistical data published by the Iranian thalassemia association, over 18,000 affected patients are living in the country. About 2500 among them are registered in the Mazandaran province. Prenatal diagnosis (PND) of thalassemia has been possible in Iran since 1992 [5]. A national prevention and treatment program for thalassemia was implemented in 1997. It is now well-established and known as one of the screening programs in the world [6].

Informative polymorphisms are important in prenatal diagnosis to differentiate between normal and mutant gene inheritance from parent to child [7]. More than 20 restriction fragment length polymorphisms (RFLPs) are known in the beta-globin gene cluster and some of them are routinely used in the PND services [8–10]. Most of these RFLPs are located out of the beta-globin gene (HBB), somewhere upstream or downstream in the beta-globin cluster [9, 10]. Some of these locations may have low allele frequency in the local population and are not informative in the prenatal diagnosis [11–14]. Besides, a hotspots point for meiotic recombination is known to be upstream of the beta-globin gene [15, 16]. Hence, this study aims to find new polymorphisms and haplotypes with high allele frequencies in the local population and, preferably, inside the beta-globin gene in order to use in linkage analysis in the prenatal diagnoses. A fairly large number of samples were sequenced and studied in this research.

## Methods

### Sample collection

A cross-sectional study on 2350 samples (1321 male and 1029 female) from the Mazandaran and Golestan provinces of Iran whom suspected to be the carriers either for alpha or beta thalassemia and referred to the local diagnostic laboratory as a routine services were investigated during five years, (2010–2015). These individuals had reduced hematological indices (MCV lower than 80 ft. and MCH lower than 27 Pg).

All peoples with microcytic and/or hypochromic condition that is tested in this study was not patient (e.g. beta-thalassemia major). Instead, the samples were included beta or alpha-thalassemia carriers as well as peoples with no mutations, because a part of the people with microcytic and hypochromic condition may not reveal any mutation in alpha/beta genes (up to 20% in our study). So the sample in this study was included affected alleles (beta-thalassemia carriers) as well as non affected alleles (beta-thalssemia carriers, alpha-thalassemia carriers and people with no mutation).

They were nominated for genetic tests as a part of the national screening program for the prevention of thalassemia [5]. All the individuals had signed consent forms for participating in this research project.

### DNA extraction and PCR amplification

A 5 to 10-ml blood sample was taken into an EDTA containing tube. The lymphocytic genomic DNA was isolated according to a previously published method [17]. Two sets of primers were used to amplify the beta-globin gene (HBB) (Table 1). Briefly, beta-globin gene was amplified by 200 mM dNTP, 1 U Taq DNA polymerase (Cinagen, Iran), 1× Taq buffer, 20 pM each primer, 1.5 mM MgCl_2_ and 1 μl (300 ng) of DNA were mixed and the volume was adjusted to 25 μl with water. Amplifying a 861 bp and a 578 bp fragment were initially denatured at 95 °C for one minute, followed by 35 cycles of denaturation at 95 °C for 60 s, annealing for 60 s, and extension at 72 °C for 60 s and a final extension at 72 °C for 5 min. The samples were then subjected to a final extension step at 72 °C for 5 min. A 6-μl aliquot of each PCR product was run on a 1% flatbed agarose gel to check for positive amplification [6, 18].

**Table 1:** Specific primers were designed for DNA sequencing of coding regions in the beta-globin gene. Besides, the primers were designed to genotyping of IVSII-74 (rs7480526) by the ARMS-PCR methods

Primer name	Primer Sequence	PCR size (bp)	Annealing temperature
HBB 1F	GTA GCA ATT TGT ACT GAT GGT ATG G		
HBB 2R	CTT CCA CAC TGA TGC AAT CAT TC	861	63^°^
HBB 3F	ATG TAT CAT GCC TCT TTG CAC C		
HBB 3R	GCA CTG ACC TCC CAC ATT CC	578	63^°^
IVSII-74 N	TTA AGT TCA TGT CAT AGG AAG GTG AG		
IVSII-74 M	TTA AGT TCA TGT CAT AGG AAG GTG AT		
IVSII-74 R	ATC ACT GTT ATT CTT TAG AAT GGT GC	589	60^°^
GAPDH F	CAT GGC CTC CAA GGA GTA AG		
GAPDH R	GGT TGA GCA CAG GGT ACT TTA	219	60^°^

### DNA sequencing and genotyping

Beta-globin gene was sequenced for all individuals as a part of the diagnostic genetic laboratory service and protocol. Two specific primer sets were applied to amplify the beta-globin gene (Table 1), followed by Sanger sequencing methods. Beside the mutation detection as normal diagnostic services, genotyping also was carried out for the five common polymorphisms including codon2 (C/T) (rs63750898), IVSII-16 (C/G) (rs63750254), IVSII-74 (G/T) (rs7480526), IVSII-81 (C/T) (rs7946748) and IVSII-666 (T/C) (rs1609812) for each individual (Table 2).

**Table 2:** Prevalence of SNP heterozygosity for five polymorphisms found among the people in the northern provinces of Iran

Polymorphic position	Accession number	Genotype frequency (%)			Allele Frequency (%)	
Codon 2 (C/T)	rs63750898	CC (64.72)	CT (31.72)	TT (3.5)	C (79.78)	T (20.22)
IVSII-16 (C/G)	rs63750254	CC (64.72)	CG (31.72)	GG (3.5)	C (79.78)	G (20.22)
IVSII-74 (G/T)	rs7480526	GG (36.5)	GT (54.71)	TT (8.8)	G (62.78)	T (37.22)
IVSII-81(C/T)	rs7946748	CC (78.68)	CT (19.47)	TT (1.81)	C (91.09)	T (8.90)
IVSII-666(T/C)	rs1609812	TT (64.72)	TC (31.72)	CC (3.5)	T (79.78)	C (20.22)

### ARMS-PCR for IVSII-74 (G > T)

**Fig. 1:**
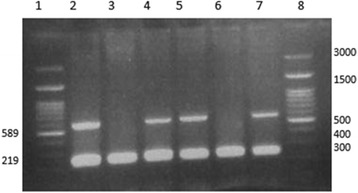
ARMS-PCR for IVSII-74 (G > T) polymorphism. Lane 2 and 3 belong to a normal individual (T/T), lane 4 and 5 belong to a heterozygous individual (T/G), lane 6 and 7 belong to a homozygous (G/G) individual, lanes 1 and 8 are a 100 bp DNA ladder

Three specific primers, including one normal (IVSII74-N), one mutant (IVSII74-M), and a common reverse primer (IVSII74-R), were designed (Table 1) in order to genotype of the IVSII-74 position. The PCR product was 589 bp long. Also a set of primers was used to amplify a DNA fragment in Glyceraldehyde 3-phosphate dehydrogenase (abbreviated as GAPDH) as an internal control (Table 1 and Fig. 1). The PCR product for internal control was 219 bp long. The following PCR conditions were applied: 95 °C for 10 min, followed by 35 cycles at 94 °C for 45 s, 60 °C for 60 s, and 72 °C for 60 s, finishing with a cycle for 10 min at 72 °C. Around 10 μl of PCR product were then run on 1% agarose gel for electrophoresis and analysis of the bands (Fig. 1).

## Results

Around 2350 individuals (1321 male and 1029 female) were studied. Five different polymorphisms in the beta-globin gene were analyzed using direct DNA sequencing method. Table 2 showed the genotype and allele frequency of five common polymorphisms in the beta-globin gene found in the northern provinces of Iran. In this study, the SNP heterozygosity was calculated separately for each five polymorphism. The codon2 (C/T) statistics showed 31.72%, IVSII-16 (C/G) 31.72%, IVSII-74 (G/T) 54.71%, IVSII-81 (C/T) 19.47%, and IVSII-666 (T/C) 31.72%, respectively. Although all five polymorphisms showed reasonably high heterozygosity, IVSII-74 (G/T) [GG wild type (36.5%), G/T (54.71%) and TT (8.8%)] showed the highest heterozygosity rate in this study (Table 2).

The genotype distribution of 5 single nucleotide polymorphisms (SNPs) was analyzed for Hardy-Weinberg equilibrium (HWE). From 5 SNPs, 4 were compatible, except IVSII-74 (G > T) which did not show compatibility with HWE.

An association of these five polymorphisms was studied as four different haplotypes, named M1 to M4, which earlier defined as “beta-globin framework” (Table 3) [13]. As many as 63.91% of alleles showed no sign of these five nucleotide changes in the beta-globin gene sequence at the indicated locations and the nucleotides were matched with haplotype M1 sequence (Table 3). The remaining alleles were M2 with 16.75%, M3 with 11.74% and M4 with 7.6% frequency respectively. Compared to M1 as the major haplotype in population, M3 haplotype has nucleotide changes in all five polymorphisms (Table 3).

**Table 3:** Frequency of four different haplotypes, named M1 to M4 investigated in this study

Haplotype	Codon 2	IVS2–16	IVS2–74	IVS2–81	IVS2–666	Frequency (%)	Framework^a^
M1	C	C	G	C	T	63.91	Framework 1
M2	C	C	T	C	T	16.75	Framework 2
M3	T	G	T	T	C	11.74	Framework 3
M4	T	G	T	C	C	7.6	Framework 3a

^a^These haplotypes was reported from the same area in Iran and were defined as frameworks by Niaki and colleagues earlier [13]

## Discussion

Two routinely-applied main approaches for prenatal diagnosis include mutation detection or the direct method and linkage analysis using polymorphic sites as a DNA marker, also known as the indirect method are normally applied in our diagnostic genetic laboratory [19]. Meanwhile, over 20 SNPs for restriction enzymes have been recognized in the beta-globin gene cluster so far [8–10]. Most of these sites have a low frequency among the Iranian population and are relatively far from the beta-globin gene [12–14, 20]. Only a few are near or inside the beta-globin gene, including the β-RsaI, β-AvaII, and β-hinfI restriction sites [21]. There is also a hot spot for recombination near the 5′ end of the beta-globin gene. So, there is a possibility of mistake in detecting mutated allele through linkage analysis [15, 16]. In order to increase the accuracy of the indirect mutation analysis, the evaluation of new SNPs and their use in the molecular analysis of beta-thalassemia are investigated in this study.

Five polymorphisms have recently been reported in the beta-globin gene in the Iranian population [13]. Akhavan Niaki and colleagues reported the RFLP restriction digestion for some of these polymorphic loci, including Codon2 (C/T) (rs63750898) recognized by HgiAI, IVSII-16 (C/G) (rs63750254) recognized by AvaII, and IVSII-666 (T/C) (rs1609812) recognized by SspI [13]. In their study of 211 Mazandarani individuals using SspI restriction enzyme in PCR-RFLP method on IVSII-666 polymorphic site, the relevant frequency of 14.3% was reported [13]. In the current study, we evaluated five common polymorphisms in the beta-globin gene in 2350 individuals. Our results indicated the frequency of 31.72% for codon 2 (C/T), 31.72% for IVSII-16 (C/G), 54.71% for IVSII-74 (G/T), 19.47% for IVSII-81 (C/T), and finally, 31.72% for IVSII-666 (T/C) respectively (Table 2). This research also indicates that the IVSII-74 (G/T) polymorphism has the highest frequency among the people from the northern parts of Iran.

Different restriction sites mainly outside the beta-globin gene (HBB) sequence are used now by a diagnostic genetic laboratory in haplotype analysis. We proposed IVSII-74 (G/T) as a polymorphism located in the HBB gene with high heterozygosity rates in Iranian population as a new haplotype marker. A simple ARMS-PCR method also has been established to genotype this polymorphism in different individuals in this study (Fig. 1).

A frequency of 4 different haplotypes as we called M1 to M4 included five different polymorphisms in the HBB gene are shown in Table 3. Niaki and her colleagues have also defined these 4 haplotypes as frameworks earlier [13]. The frequency of the haplotypes achieved in this study was different with those reported earlier from the same area because of the sample sizes. The sample size in this study was 2350 individuals from the general population compared with 46 thalassemia patients investigated before [13]. Haplotype M1, which has no nucleotide changes in any five polymorphic sites, registered the highest frequency at 63.91% and the haplotype M4 with nucleotide change in 4 different polymorphic positions out of 5, registered the lowest at 7.6% (Table 3).

This research introduced a new intragenic position which can be used along with previously known markers in the PND diagnostic services. Also, along with IVSII-74, other intragenic polymorphisms such as IVSII-81 with high heterozygosity rate (19.47%) in the northern population of Iran could prove to be better candidate markers for the prenatal analysis compared with the previously known markers outside the HBB gene.

## Conclusion

This is the first study with a relatively large number of participants (2350 individuals) on the beta-globin gene polymorphisms in the Mazandaran and Golestan provinces of Iran. Using IVSII-74 polymorphism with high heterozygosity along with the previously known markers in PND services is recommended.

## Abbreviations

GAPDH: Glyceraldehyde 3-phosphate dehydrogenase
GO-MOH: Genetics Office in Iran’s Ministry of Health and Medical Education
HBB: beta-globin gene
PND: Prenatal diagnosis
RFLPs: Restriction fragment length polymorphisms
